# The Nursing Supplement

**Published:** 1890-02-15

**Authors:** 


Being the Extra Nursing Supplement of "The Hospital" Newspaper.

All Contributions for this Supplement should be addressed to the Editor, The Hospital, 140, Strand, London, W.C., and should have the word "Nursing" plainly written in left-hand top corner of the envelope.

### En Passant.

Coventry District Association.—Lord Leigh presided over the annual meeting of this association. The Misses Wing continue to do good work as nurses among the poor, and the financial position is satisfactory. Miss Squirrel, who trained at Coventry Hospital, has joined the Misses Wing in their good work.

Nurses' Co-operation.—We have received the following letter: "May I by means of your interesting paper tell the private nurses how pleased I am that they are at last starting an association to receive their own earnings? It is peculiarly aggravating to feel that your own dear nurse who for weeks has spared neither thought nor fatigue for your comfort and recovery, and for whose services you are probably paying two guineas per week, gets really a pittance of £25 per year or less. My only wonder is that so large a body of trained women have not established their own offices before. I am sure there are many private patients who think with me." We also are glad to learn that this movement is taking substantial form, and that a central office will shortly be opened under excellent management.

Thousands of Pounds for the National Pension Fund.—We have the pleasure to acknowledge the following sums towards the £14,000 now being raised to at once bring the Donation Bonus Fund up to £40,000. We hope to be able to acknowledge contributions amounting to £1,000 every week until the £40,000 are completed. Contributions of any amount may be sent to the Editor of The Hospital, or to the Manager, National Pension Fund for Nurses, 8, King Street, Cheapside, London, E.C.:

The Fourth Thousand.

| | £ | s. | d. |
|---|---|---|---|
| Messrs. Morris, Hurst, and Puckle ... | 105 | 0 | 0 |
| Mr. C. C. Gowan ... ... ... ... | 100 | 0 | 0 |
| Messrs. Greenwell and Co. ... ... | 100 | 0 | 0 |
| Mr. John S. Scrimgeour ... ... ... | 100 | 0 | 0 |
| Mr. Herbert Leon... ... ... ... | 100 | 0 | 0 |
| Mr. Sydney Kennedy ... ... ... | 52 | 10 | 0 |
| Messrs. Seligman Bros. ... ... ... | 50 | 0 | 0 |
| Mr. E. De la Penha ... ... ... | 50 | 0 | 0 |
| Mr. T. Nickalls ... ... ... ... | 50 | 0 | 0 |
| Messrs. Oates and Cockburn ... ... | 26 | 5 | 0 |
| Messrs. Rickardo, Poston, and Co. ... | 26 | 5 | 0 |
| Messrs. Hopkins and Langton ... ... | 25 | 0 | 0 |
| Messrs. H. Rickardo and Co. ... ... | 25 | 0 | 0 |
| Messrs. Kennedy and Robertson ... | 21 | 0 | 0 |
| Messrs. Midlane and Co.... ... ... | 21 | 0 | 0 |
| Messrs. Gordon, Lawford, and Co. ... | 21 | 0 | 0 |
| Messrs. Laing and Fletcher ... ... | 10 | 10 | 0 |
| Messrs. Nickisson, Evans, and Co. ... | 10 | 10 | 0 |
| Messrs. Hollebone Bros. and Trench ... | 10 | 10 | 0 |
| Messrs. White and Cheeseman ... ... | 10 | 10 | 0 |
| Messrs. Symons and Posen ... ... | 10 | 10 | 0 |
| Messrs. R. O. Nivison and Co. ... ... | 10 | 10 | 0 |
| Messrs. Hyam Bros. ... ... ... | 10 | 10 | 0 |
| Messrs. G. Jacobson and Sons ... ... | 10 | 10 | 0 |
| Mr. P. Nickalls ... ... ... ... | 10 | 10 | 0 |
| Mr. J. N. Scott ... ... ... ... | 5 | 5 | 0 |
| Messrs. Walford Bros. ... ... ... | 5 | 5 | 0 |
| Mr. J. P. Kitchin... ... ... ... | 5 | 5 | 0 |
| Messrs. Philpot and Clarke ... ... | 5 | 5 | 0 |
| Mr. Jeffery Whitehead ... ... ... | 5 | 5 | 0 |
| Messrs. W. Morris and Son ... ... | 5 | 5 | 0 |
| Mr. E. Swift ... ... ... ... | 1 | 1 | 0 |

Amount already acknowledged, **Four Thousand Pounds.**

Sunderland Nursing Institute.—The Mayor presided over the first annual meeting of this institute, which was opened in December, 1888. Sister Kathleen, late of the infirmary, is district nurse among the poor. Miss Jeffery, also trained at Sunderland Infirmary, is matron, and has 14 nurses under her. The institute has been so largely appreciated that many applications for nurses have had to be refused.

Workhouse Infirmary Association.—Once more we record the progress of this excellent association, which has just issued its tenth annual report. The income of the association has never exceeded £250, and yet it has supplied 288 nurses and looked after their material and moral welfare. During last year 89 applications were received from boards of guardians, but only 53 nurses could be supplied, of whom 19 were trained by the society. The office at 6, Adam Street, Strand, is now open daily from eleven till one.

Kent and Canterbury Institute.—The number of nurses at the beginning of the year was, exclusive of the district nurse, nine; at the beginning of 1890 it will be, exclusive of the district nurse and the maternity nurse, fourteen, and there are four probationers in training at the hospital. One hundred and thirty-three cases of paying patients have been attended in 1889 as against seventy-six in 1888. The institute has a balance of about £200 at the bank. Miss Wilks retired from her post of lady superintendent last year, and was presented with a purse of money in recognition of her services.

Short Items.—We learn that Mr. Wilson, of Wimpole Street, does not train male nurses, he only supplies them.—Miss Clara Jump now signs herself Sister Faith.—At Cardiff Union Hospital night-nursing has been started, and the abuses connected with the employment of untrained nurses and paupers have been got rid of.—We call the attention of midwives and monthly nurses to a case noted in last week's *British Medical Journal* in which a nurse infected her patient, a confinement case, with scarlet fever.—Mdme. Andouard, who has lately died at Nice, was a devoted hospital nurse during the siege of Paris.

Registration.—We have before us the papers just issued by the British Nurses' Association regarding their register of nurses. There is a significant note on Form 2—"If no certificate is held by applicant, explain why." Of course, those nurses who do hold certificates can get no benefit from the register, and it is just those nurses who have not got certificates who will pay their 10s. 6d. in hope of obtaining some advantage in return. Probably they will think a British Nurses' Association certificate is better than none, but even in this they may be mistaken. We have published the names of the six out of the 200 or more hospitals of Great Britain and Ireland, who alone approve of this scheme of registration, and now we leave it to the common sense of nurses either to side with the 200 or with the 6, according as they think it will be most to their advantage. We would also advise nurses to consider such a pledge as the following before signing it: "I promise in the event of my being so registered, and in consideration thereof, to be bound by, and so conform in all respects to the rules and regulations for registration for the time being in force. And I undertake to return my certificate of registration to the registrar, if I am called upon to do so by the Registration Board."

### Nursing Lectures.

By Percy Lewis, M.D.

### Lecture II.—CHEST DISEASES.

We will begin this course with the nursing of chest cases, and, so first of all—What is the chest? Well, the chest is the upper part, almost the upper half, of the trunk which contains the organs of respiration and circulation. What are the uses of respiration and circulation? Briefly, respiration is the process by which air gets into the chest and so into the blood; the circulation of the blood carries food and air to, and removes waste products from, the different parts of |the body.

Air is a mixture of three gases, oxygen, nitrogen, and carbonic acid; oxygen being the most important, for without it we could not exist. Nitrogen simply dilutes the oxygen which, like spirit, is too strong to be taken undiluted. Carbonic acid gas in ordinary air is in very minute proportion. There is always more or less watery vapour mixed with the air. By respiration men and animals give out carbonic acid and absorb oxygen; should the supply of oxygen be cut off from any cause, death inevitably results. The consequence directly or indirectly of most chest diseases is to limit the means by which air, *i.e.*, oxygen gets into the blood, and finally, perhaps, to cut it off altogether, hence you will at once see how important this branch of your subject is.

Very briefly indeed I will mention the rough anatomy of the chest and its contained organs. The chest is an elastic box, of the shape of which you will be able to get some idea by looking at a skeleton. You see that it is formed almost entirely of ribs, which are attached behind to the spine and in front by means of short elastic pieces of gristle (cartilages) to the sternum, or breast-bone. It is not open at the bottom as you see it in the skeleton, but is closed by a dome-shaped muscle called the "diaphragm," concave towards the abdomen and convex on the chest side. It is attached to the inner sides of the lower ribs and their cartilages, extending up in front to the lower end of the breastbone and backwards to the spine. This is a most important muscle of respiration, as by its contraction the capacity of the chest is alternately increased or diminished. The upper opening of the chest is closed by the tops of the lungs and by the structures entering the chest from the neck. The ribs are joined together by little muscles called intercostals, and are covered on the outside by various large muscles, mostly those used in moving the arm, head, or spine.

The organs of respiration contained in the chest are part of the windpipe, with its many branches, and the two lungs. The windpipe (trachea) leads from the larynx, or voice box, into the chest, where it at once divides into two smaller tubes (bronchi), one for each lung, and these again and again divide until they become almost microscopic, when they leave off dividing, each expanding into a little bladder-like structure called an air cell. The ending of these small bronchi, in fact, resembles one of those squeaking toys which you see boys in the street with, consisting of a small bladder on the end of a short hollow tube with a reed in it. They blow out the bladder, which, as it collapses, gives rise to a squeaking noise. Each of these little air cells is filled with air and covered on the outside with very minute blood vessels. It is by the accumulation of thousands of these little air cells together that the lungs are formed.

The windpipe, as felt in the neck, is a more or less rigid tube; the very small bronchi I have been describing to you, are almost entirely muscular tubes capable of contracting or expanding so as to allow more or less air to enter into air cells, *i.e.*, the lungs. It is in the air cells that the exchange between the blood and the air takes place, the air giving up oxygen, receiving instead carbonic acid and watery vapour with varying amounts of excrementitious matter, which, when in excess, gives that peculiarly offensive odour to the breath in some patients so marked.

The lungs are covered on the outside with a membrane called the pleura. This consists of two shining smooth surfaces, one of which covers the outside of the lung, and the other the inside of the chest wall. The two surfaces being united at their edges form a closed bag, the sides of which are in contact. The object of the pleura is to allow the lung to move freely against the chest wall in breathing. It is lubricated by a little watery fluid which it manufactures itself. There is of course a separate pleura for each lung. In shape, the lungs are somewhat conical, they fill up most of the cavity of the chest, one occupying each half. From a nursing point of view wherever you find a rib there is also lung underneath it, consequently you will see from the skeleton how far down they extend behind, a point beginners are always long in appreciating. The lungs meet in the middle line in front behind the sternurn, except where the heart separates them, and roughly speaking, their lower edges follow margins of the ribs and their cartilages obliquely in the spine, without however extending quite so far down. Above they extend up an inch or more beyond the collar bone, which explains the bulging you will often notice in this region during an attack of coughing.

The heart, is the muscular pump which by alternately contracting and relaxing, keeps up the circulation of the blood. It contains four compartments, two for receiving blood—auricles, and two for propelling blood—ventricles. In order to make the blood go in a definite direction, valves are placed in it. You need only know the names of the two most important, the mitral and the aortic. The blood is taken to the different organs by vessels called "arteries," distributed to the component parts of those organs by minute vessels called "capillaries," and brought back again to the heart by vessels called "veins." In order that the heart may contract and expand without friction, it, like the lungs, is covered with a structure called the pericardium, having an arrangement and uses similar to those of the pleura.

The position of the heart may be represented by the closed fist of the left hand laid across the front of the chest, so placed that the little finger rests exactly on the lower end of the breast-bone; the knuckles will then only project slightly to the right of the middle line, most of the hand being to the left of it. The extreme outer limit on the left side can be ascertained by noticing in the space between the fifth and sixth ribs the point where the beating of the heart ceases to be felt. This is called the "apex," the upper limit of the heart being called the "base."

There is one other organ in the chest you should know the name of, that is the great artery of the body—the aorta. This arches back from the base of the heart over the division of the trachea, to reach the spine in contact with which it is continued down into the abdomen.

It may interest you to mention what the common chest diseases are. Now, I told you that the smaller air tubes were composed chiefly of muscle which could regulate the amount of air going to the lungs. From various circumstances these sometimes contract too much, causing the patient to become half-suffocated, and to fight and struggle for breath; that is asthma. The tubes may become inflamed, and filled with secretion—bronchitis; or dilated, secretion accumulating and putrifying in them—bronchiectasis. The air cells may become dilated and thickened, so that the whole lung is larger and less useful—emphysema; the air-cells may become filled with a solid exudation from the blood, changing the lung tissue from its natural consistence of a sponge to that of a piece of liver, and thus rendering a large portion of it useless—pneumonia; congestion is an early stage of pneumonia. Small portions of the lungs may be affected in this way, subsequently breaking down to form cavities, and being coughed up with varying amounts of secretion—phthisis or consumption. Large portions of the lungs may die and putrefy—gangrene. The pleura may get inflamed and filled with fluid—pleurisy with effusion. This fluid may be converted into matter or pus as it is called—empyœma. Air may get into the pleura—pneumothorax.

Heart disease most often means disease of the valves, either narrowing them—stenosis—or widening them, allowing blood to partially flow the wrong way—regurgitation. The disease is called after the valve affected, *e.g.*, a patient is said to have "aortic regurgitation," or "mitral stenosis." The pericardium may become inflamed and perhaps filled with fluid—pericarditis with effusion.

The aorta may from disease become weak in one part, and then the pressure of the blood inside expands it more and more until it presses on neighbouring organs, interferes with their functions, and finally, perhaps bursts—aneurism.

### Notes from Australia.

*(By Our Own Correspondent.)*

Melbourne, December 20th.

The excitement of the month has been the election of a matron to the Melbourne Hospital. There were twenty-two applications for the appointment, and this number, by rough selection, was reduced to eight, as follows: Miss Isabella J. Rathie, Hobart Hospital, Tasmania; Miss Marie Herzog, hospital, Bathurst, N.S.W.; Miss Jessie L. Davey, Nurses' Home, Sydney; Miss Sarah Bryce, South Yarra; Miss Isabella Ashby, private hospital, Creswick; Miss Betsy Irewhella, matron, Kyneton Hospital; Mrs. Kate M. Watson, late matron of Geelong, Ballarat, and Homœopathic Hospitals; and Mrs. Isabella Rice, trained in Royal Southern Hospital, Liverpool. Miss Rathie was elected, and she will have difficult work to get a decent nursing system started. If she is wise, she will get some nursing sisters from England to help her. The election was not concluded without a row, and Mr. Williams is still in disfavour with the nurses, so that the new matron is not to be envied.

At the Medical Hall, Albert Street, on December 11th, Professor Allen, who is about to pay a prolonged visit to Europe, was presented with a handsome bookcase, a service of silver plate, and an illuminated address by the members of the Victorian section of the Intercolonial Medical Congress, in recognition of his services as honorary secretary of the reunion of 1889. The president of the congress (Mr. T. N. Fitzgerald) occupied the chair, and there was a large gathering of the leading members of the medical profession.

The Australian Health Society has been giving its usual course of interesting lectures. At their annual meeting, the secretary reported that the course of public lectures just concluded had been well attended, the audiences numbering from about 150 to 400; that the new volume of "Health Lectures for the People" and the fourteenth annual report had been sent to all the members; that nine new subscribers had been enrolled since the previous meeting; that the last edition of No. 8 tract, entitled "Diseases which should be Prevented," by Dr. Jamieson, was exhausted: and that various additions had been made to the library.

At the committee meeting of the Women's Hospital, a letter was read from Dr. Felix Meyer with reference to a report made by him to the committee to the effect that the administration of the midwifery department was eminently unsatisfactory, for the reasons that there was unaccountable delay in dealing with cases, that the linen was not fit for use, and that there was no proper supervision over the nurses. A sub-committee, which had been appointed to inquire into the matter, reported that, having carefully investigated the complaint, they found that it was wholly unsustained, and that Dr. Meyer was deserving of blame for having preferred so grave and emphatic a complaint upon such insufficient grounds. Still, privately it is said that there is no smoke without some fire.

The District Nursing Society is raising funds for building a central home for its nurses.

The first electric tramway in Australia was opened here last month on the hilly Doncaster route. It has so far been a great success.

The new convalescent home for women, about fifteen miles from the city, was opened in November by Lady Loch, and is doing well.

The annual report of charities, drawn up by Captain Evans, is before the Government. Among those things which meet with Captain Evans's warmest approval may be mentioned the boarding-out system, which has been largely adopted both by the Government and by private philanthropic societies in dealing with neglected children. The inspector of charities desires to see it still more extensively tried by the managers of orphanages, partly because his experience has driven him to the conclusion that a home life is preferable to that of an institution, and partly because the children who are sent to the country take to rural pursuits, and are soon able to provide for themselves.

One delirious patient having killed another at the City Hospital, it has been discovered that a single nurse had charge of six patients all suffering from delirium tremens. Dr. Youl says the refractory ward was condemned by a jury ten years ago, but still exists in the same unsatisfactory state.

I have been going through the slums by daylight. Nine women, in various stages of drunkenness, were in one of the lanes at four o'clock one morning. One was laid on the pavement asleep; another, young and well dressed, reeled from side to side, catching hold of a lamp-post or the wall as she went along. It was a sickening sight, and I felt very hopeless of doing any good with such material. The mission woman I go with has been working in the neighbourhood for nine years. Her patient persistence is wonderful, and it is quite a relief to me to hear now and then a strong man or woman say "Well, mother, how are you?" in a tone which says, "My life is much nobler and better for your loving care, and I thank you for it."

During this month I went one day to the Eye and Ear Hospital. It is one of the most efficient institutions in Melbourne, and does its work with quiet unobtrusiveness. Mrs. Wylie is the matron—a post she has held for eighteen years. The building is conveniently situated on the north side of the city.

### Everybody's Opinion.

*[Correspondence on all subjects is invited, but we cannot in any way be responsible for the opinions expressed by our correspondents. No communications can be entertained if the name and address of the correspondent is not given, or unless one side of the paper only be written on.]*

### THE TITLE OF NURSE.

A Nurse writes: I have read the discussion on Miss Broadwood's paper with great interest. It seeme to me that we are getting involved as to the meaning of the word "nurse." By all means let "trained nurses" be trained nurses, but let the adjective do its own work, and as long as the English language is our means of expression, let a nurse be "one who has the care of infants," and "one who tends the sick." (Nuttall.) Surely that woman who goes to "tend the sick" in cottage homes has an equal right to the title "nurse" with the most highly-trained hospital nurse in the land. In our work (nursing the poor in their own homes) we come across innumerable "nurses," humble, handy, sympathetic, unselfish women tending sick neighbours, often and often for no hope of reward; we never deny them their title of nurse. Surely they are just as much nurses as we are, and I for one should be sorry if it were decided to call them "helps," and leave us alone with the name of "nurse." It is our common right.

### NURSING IN AMERICA.

Mrs. L., Albany, writes:—By the time this reaches you the long-hour controversy will probably be at an end, but still I feel I should like some of my English sisters to know how things are on this side of the Atlantic. I am an English trained nurse (London), and have held various appointments in English hospitals; am now holding one in an American one, where the hours are from six a.m. to seven p.m. with no off duty, except a fortnight once a year. It is quite impossible to attend a church, except during that fortnight, and equally impossible to make any acquaintances. There is no system whatever in the hospital, and a nurse who is at all conscientious spends her life one perpetual grind; others who take no interest whatever in their work appear to get the best of it in every way; trained and untrained all are on a level. The salary is about £45 per year in English money, but everything is so expensive that I found myself much better off in England with £30. I should like to give you some details of a nurse's life here, but it would make my letter too long, and I fear you would hardly believe them. One thing I must say, and that is, as regards a nurse's social position here, it is infinitely inferior to that in England. I attribute it to the fact that the majority of the doctors are not gentlemen. Anyone that can scrape the money together by any means can get through college here and graduate. I know several who hire out between terms as waiters, coachmen, clerks, &c. When they get through they are so frightfully conceited that there is positively no living with them; and a nurse in their eyes occupies a position in which they may possibly keep themselves respectable, but it is very improbable, and certainly it is no fault of the doctor if they do. The asylums here give their attendants—or, as they call them, "nurses"—two years' training, and at the end of that time a diploma. That is all very well, but I fail to see why they should be received into hospitals as trained nurses when they do not know how to take a temperature, or even to make a poultice. Private nursing pays very well here: a good nurse can get pretty constant work at $20 per week, but her board, lodging, washing, &c., will cost her about four times as much as in England. Take it all in all, I would strongly advise the English nurses to stay at home. I, for one, am very sorry I came out; but before giving up I propose trying farther West. At present this is the only hospital I have been in out here.

### Appointments.

Hope Infirmary.—Miss J. B. Mayhew, who was trained at Guy's, and was for three years matron of the Brecon Hospital, has been appointed night superintendent of the Hope Hospital at Eccles.

Cardiff Union.—The many friends of Miss Mewburn, night sister at the Glamorganshire and Monmouthshire Infirmary, Cardiff, will be pleased to hear of her appointment as Nurse Superintendent to the Cardiff Union Hospital, an institution containing nearly 300 beds, and the Cardiff Guardians are to be congratulated at securing the services of such an excellent and so efficient a nurse. Miss Mewburn trained at Leeds, was staff-nurse at Dudley, sister at Cardiff Infirmary, and night superintendent at Marylebone Infirmary. Her rise has been steady but sure, and we wish her all success in her new post.

Miss Foggo-Thomson.—Miss L. E. Burgess writes from Truro: "I think it is only fair to Miss Helen Foggo-Thomson to say (whether she holds a certificate or not) that she is a very clever and practical lecturer. She had a very large class here last spring, and gave universal satisfaction. I was present at some of her lectures, so can speak from experience."

### Death in our Ranks.

We regret to record the death (on January 27th, at Derby) of Nurse Gwendoline Ashley, who, whilst nursing a case of scarlet fever, contracted the disease herself. Rheumatic fever followed, and after a lingering and trying illness she entered into rest on her twenty-ninth birthday.

### Notes and Queries.

### Queries.

(51) *Nurses for Nurses.*—Is it usual for a nursing home to charge an old member of the surgical staff full fees for supplying a nurse?—*A. S.*

### Answers.

*N. H. D., Nurse L., and others.*—We have written to ascertain the price of the book on Obstetric Nursing. Drs. Haultani and Ferguson's "Handbook of Obstetric Nursing" is to be obtained from the publisher, Mr. Young J. Pentland, 11, Teviot Place, Edinburgh, post free for 4s. 1d.

*Testimonials.*—You can have them printed at Messrs. Collingridge, *City Press*, 148, Aldersgate Street, London, E.C.

*Gretta.*—There are so many institutions for teaching employment to the blind that we must refer you to Burdett's "Help in Sickness" for full particulars. There are workshops for the blind of Kent at Greenwich (1, South Street), which we think would meet your case.

*M. D.* is requested to send name and address.

### Notices.

Exhibition of Nursing Uniforms.—This exhibition will be on view at the Salop Infirmary, on February 18th, 19th, and 20th, from two till five p.m. Admission 1s., all nurses in uniform free.

Hollond Institution, Nice.—The Directress begs to state she cannot answer all the very numerous letters she received in answer to her advertisement in The Hospital, and, therefore, has only written to those who seemed most suitable.

### Vacancies.

The following vacancies are announced:

### Matrons.

Caterham Cottage Hospital; £30.

Croydon Hospital; £60.

Stockport Infirmary; £60.

St. Olave's Union; £100.

West Bromwich Hospital; £50.

### Sister.

Monsall Fever Hospital, £32.

### Nurses.

Aimee Nursing Home.
Arbroath Poorhouse; £25.
Bournemouth Institution; £25.
Bournemouth Victoria Home; £25.
Belgrave Hospital for Children.
Beckell Hospital, Barnsley; £20.
Beresford House, Beaumont Street.
Bongor Nursing Institute; £25.
Blackheath Institute.
Buckinghamshire General Infirmary; £25.
Bicester Union; £24.
Brixton and South London Association of Nurses.
Chester Deaconess House; £24.
Church Deaconess' House, Chester; £24.
Eye and Ear Infirmary, Liverpool; £20.
Fitzroy House, Fitzroy Square; £25.
General Nursing Institute.
Hanover Institute, 22, George Street, Hanover Square; £25.
Jersey Nurses' Institute; £25.
King's Cross Hospital, Dundee; £24.
Leicester Institution; £25.
Montreal, Canada; £37.
Mr. Wilson's Institution.
Newcastle Nurses' Home; £22.
Nottingham Nursing Association; £25.
Newport Nursing Institute.
Pendlebury Hospital; £20.
Paris Levick Institution.
Staffordshire Nurses' Institution, Stoke-on-Trent.
St. Vanda's Nursing Home, Bromley.
St. Saviour's Infirmary, East Dulwich Grove; £20.
St. Cross Hospital, Rugby; £25.
Shoreditch Infirmary; £20.
Sheffield Borough Hospital; £25.
St. Peter's, Bedford; £26.
St. Saviour's Infirmary; £20.
St. Leonard's Nurses' Home.
Swansea Nursing Institute; £25.
Tunbridge Wells Hospital; £25.
Torquay Nurses' Institution.
Wirrall Children's Hospital; £23.
Wimbledon Convalescent Hospital; £22.
Weston-super-Mare Institute.
Whitechapel Infirmary; £20.
West London Hospital; £28.
Wimbledon Convalescent Hospital; £25.
Yeovil Hospital; £30.

### District Nurses.

Bristol District Society.
Bedwyn, Wilts; £25.
Maindy, Cardiff; £30.
Southport Institution (midwife).

### Probationers.

Batley Cottage Hospital.

Bolton Infirmary.

Great Yarmouth Hospital.

Hospital for Women, Lupus Street.

Kendal Hospital; £10.

Kent Nursing Institution.

Princess Alice Memorial Hospital, Eastbourne.

Nottingham Hospital for Women.

Nottingham Nursing Association.

Workhouse Infirmary Association.

### Amusements and Relaxation.

### FOURTH QUARTERLY WORD COMPETITION

Commenced Jan. 4th, 1890, ends March 29th, 1890.

Three prizes of 15s., 10s., 5s., will be given for the largest number of words derived from the words set for dissection.

Proper names, abbreviations, foreign words, words of less than four letters, and repetitions are barred; plurals, and past and present participles of verbs, are allowed. Nuttall's *Standard* dictionary only to be used.

N.B.—Word dissections must be sent in WEEKLY, arranged alphabetically, with correct total affixed.

The word for dissection for this, the SEVENTH week of the quarter, being

"APPLES."

| Names. | Feb. 6. | Totals. | Names. | Feb. 6. | Totals. |
|---|---|---|---|---|---|
| Holland | 47 | 256 | Nurse Clague | — | 40 |
| Red Cape | — | 132 | Lauriston | 46 | 285 |
| Nurse Marie | 31 | 195 | B. E. T. | 34 | 175 |
| Garfield | 43 | 243 | Jacko | — | 110 |
| Midget | 28 | 236 | Lady Clara | 39 | 258 |
| E. M. S. | — | 149 | Patience | 48 | 287 |
| Sister | 44 | 247 | Ecila | 48 | 276 |
| Duchess | 29 | 115 | Maude | 48 | 278 |
| Ellice | 46 | 273 | Esperance | 48 | 282 |
| Reldas | 46 | 283 | Ruby | 43 | 273 |
| Jenny Wren | 44 | 267 | M. W. | 48 | 282 |
| Mopus | — | 122 | Crystal Palace | 46 | 228 |
| Neptune | — | 118 | Jackanapes | 32 | 191 |
| Qu'appelle | 46 | 266 | M. M. M. | — | 25 |
| Whitehoe | 43 | 254 | A. F. | 30 | 30 |

### Notice to Correspondents.

N.B.—All letters referring to this page which do not arrive at 140, Strand, London, W.C., by the *first post on Thursdays*, and are not addressed PRIZE EDITOR, will in future be disqualified and disregarded.

